# A Hundred Years before He Is Born

**Published:** 1917-07

**Authors:** 


### A HUNDRED YEARS BEFORE HE IS BORN.

When persons holding positions of authority, and presumed to be scientifically informed, make dogmatic stattments, the public comes to believe those statements because of their authority. Under such circumstances it becomes extremely difficult to give new information on the subject in any other way than by making a direct attack upon the dogma, and the lack of scientific knowledge on the part of those who give out such dogmas as science.

In a recent book is found the following:

"A couple of generations ago it was possible to teach that a child's education should begin 'a hundred years before he is born' for we could then believe that the education of parents actually affected the characters which they transmitted to their offspring. But with the new knowledge of heredity such belief is no longer possible, since education is nothing but a series of acquired characters and hence not transmitted by inheritance. The education of the parents has no effect upon the inherited traits of the children.''

The above quotations is a good example of what has become chronic among the superficially informed. Instead of investigating the effects of educations itself, they investigate some unrelated matter and then draw unwarranted conclusions. The statement that acquired characters, more properly called acquirements, are not inherited has no foundation in fact, and was never investigated by any of those persons who made the statement. Acquirements are always inherited, and such inheritance is the sole means by which we get evolution from a lower to a higher stage. There is an abundance of evidence bearing on that fact, and the accuracy of that evidence has never been questioned.

The error into which biologists fall in this matter is based on superficial consideration and a failure to distinguish in several important matters. The verb 'to acquire' is an active verb and implies direct action or the performance of work on the part of those which do the acquiring. Getting an education requires mental work, and the acquirement is a mental development coming as the result of that work. The amount of mental development which a person acquires as the result of getting an education is represented by the amount of work performed. None of those persons who tell us that acquirements are not inherited ever measured up an acquirement of any kind and then compared the amount of the acquirement with the inheritance of the subsequently produced offspring.

They have not even investigated acquirements for one generation, when any investigation of the matter to be reliable should be extended for three generations. If a man's education should begin ''a hundred years before he is born,'' then it should begin with his great grandparents. A man has two parents, four grandparents, and eight great grandparents, a total of fourteen individuals. When the biologist has carefully measured up the amount of education acquired by each one of these fourteen persons before reproducing, and then compared those measurements with what is the normal acquirements of the average parent when the average child is born, and also compared the final product with the great-grandparents, it will be time to pay some attention to his statements. Until he does do something of that kind his statements may be dismissed as unworthy of consideration.

There have been, in this world, many eminent men of intellectual power. In the matter of mental power they had "old heads on young shoulders." The "old head" is one educated by experience and developed by mental activity. Augustus, Bacon and Humboldt had old heads on young shoulders in the sense that they had great mental power in organs which were relatively young in years. They had these old heads because they were sons and grandsons of old heads. It was not necessary for them to work hard mentally to get all the mental power they had. Their fathers and grandfathers did the greater part of the mental work for them, and then transmitted the acquired development to their sons.

Octavian, surnamed Augustus bacause of his great wisdom, was the man who changed the Roman Republic into an Empire, after that republic ,was established for more than three times as long as the United States has been established. And he did it " in a manner which has no parallel in history." Augustus came from three generations of "old heads" extending back more than one hundred and seventy years, or an average of about fifty-seven years from father to son. Descent from such old heads for three generations is among the rarest of rare things. And that rare combination made the equally rare Augustus.

Bacon was also the son of an old head, and one which had received an education at Cambridge in its youth. The father was fifty-two years of age when Francis Bacon was born, and that father was himself the son of an old head. The mother of Bacon was an intensely educated woman, and was the daughter of an old head.

Alexander Von Humboldt was also the son of an old and highly educated head. His father was a major in the Prussian army, and had been raised to the position of royal chamberlain for his strenuous services. The head was forty-nine years old when Alexander was born.

These are mere examples of a thing which is universally true. A man of great intellectual ability is always the product of an education which began ''a hundred years before he was born.'' None of them was ever produced in any other way, and breeding operations which fail to educate the ancestors always results in producing the mediocre or the feeble-minded. This lack of education may come from the lack of schooling and lack of mental activity, or it may come by using young heads for parents—head which have not had time to become well educated.

Benjamin Franklin was the son of an old head, and his father was the son of an old head, and so was the grandfather and other ancestors for five generations in succession. And his mother was the daughter of an old head. But Benjamin had a son when he was a young head, and do what he would, he could make nothing more than an ordinary man out of that son.

Peter the Great was the son of an old head. When Peter was a young head he had a son, but that son had none of the ability of his father. Louis XIV was the ''Great'' King of France. He was from two generations of old heads. But from Louis XIV on, the descent was from young heads, and mental ability disappeared entirely from those descendants.

And so it always is. If we give each generation an increased amount of education before reproducing, the race will advance one step. If we give each generation a decreasing amount of education before reproducing, the race will decline step by step to a lower level of intelligence.

Practically all states in the United States permit minors to marry, and many states permit a fourteen-year-old boy to marry a twelve-year-old girl. Minors marry and minors have children. Two or three generations of such reproduction results in starting a new feeble-minded family into existence, and these feeble minded families form one of the difficult problems of society. The sure road to the elimination of the feeble-minded is the extended education of children and the prevention of marriages by minors. It is the scientific remedy because it strikes directly at the original cause of feeble-mindedness.

That superior men and women, as a class, come from mature parentage extending over two or three generations is a fact which is now acknowledged by every one who has made any attempt to investigate the matter. Those who deny the inheritance of the acquirements do not, however, investigate the actual education involved, but attempt to dismiss the matter by guesses which they offer as scientific explanations. In considering such guesses the reader should take note of the fact that any explanation offered, to be sound, must apply to the trotting horse just as well as to human beings.

In one hundred years the American trotter was developed from a three-minute trotter to a two-minute trotter. From the two-minute trotter to to-day back to the ancestor of one hundred years ago, there is a trifle over seven generations. As a consequence, the two-minute trotter has in that period more than two hundred and fifty progenitors. Those progenitors were mature to the extent of the entire number of them averaging more than four years above the average age of sires and dams. That means more than a thousand years of excess age piled up in one pedigree to get a two-minute trotter. Those "years" involved in producing improved trotters need explaining just as much as the years in human beings.

There is also another angle to the trotting horse which goes directly to the question of "educating the grandfather." The mere piling up of years in the trotting parent, unaccompanied by actual education in trotting, never produced any improvement in the trotting stock during the nineteenth century. The two hundred and fifty odd progenitors in the pedigree of a two-minute trotter were horses which actually worked hard at the trot for years before reproducing. Every attempt to improve the trotter by selection and breeding, without strenuous and long continued trotting education, resulted in failure, and horse-breeding history is full of the records of such failures.

These facts are facts of record, and the biologist who does not know them is willfully ignorant. If he feels disposed to deny them off-hand he might take note of the fact that the records are of a kind which anyone can investigate for himself. All that it is necessary to do is to write out pedigrees with dates and examine published history.—Casper L. Redfield, Chicago, Ill., The Medical Times.

